# Healthy Body and Mind Program to Improve Health Outcomes and Reduce Dementia Risk in People With Osteoarthritis: Protocol for a Feasibility and Acceptability Pilot Randomized Controlled Trial

**DOI:** 10.2196/75816

**Published:** 2025-11-06

**Authors:** Claire V Burley, William Yeates, Kelly A McLeod, Matthew D Jones, Nattai Borges, Henry Brodaty, Belinda J Parmenter

**Affiliations:** 1 Dementia Centre of Excellence, enAble Institute, Curtin University Perth Australia; 2 School of Health Sciences, University of New South Wales Sydney Sydney Australia; 3 Hammond Innovations Hammondville Australia; 4 Centre for Pain IMPACT, Neuroscience Research Australia Sydney Australia; 5 Centre for Healthy Brain Ageing, University of New South Wales Sydney Sydney Australia; 6 School of Health, University of the Sunshine Coast Sippy Downs Australia

**Keywords:** healthy aging, osteoarthritis, pilot randomized controlled trial, dementia, lifestyle, physical activity

## Abstract

**Background:**

Globally, approximately 55 million people are living with dementia. Osteoarthritis occurs in half of all older adults and is associated with pain, depression, and increased dementia risk. Currently, no program exists for people with osteoarthritis and cognitive decline that addresses modifiable risk factors, such as physical inactivity and lifestyle behaviors.

**Objective:**

This study aims to examine the feasibility, acceptability, and health outcomes following the 12-week Healthy Body and Mind Program, designed for people with osteoarthritis who are experiencing cognitive decline. The findings will inform the designs of larger trials.

**Methods:**

In total, 20 consenting participants aged 45 years or older with osteoarthritis and cognitive decline will be randomly allocated to the 12-week program or a waitlist control group. Feasibility and acceptability will be assessed based on retention and adherence rates to the program, the number of people who provide consent to take part in the study, and evaluation questions following completion of the program. Health outcomes will include quality of life, cognition (global, attention, learning, and memory), pain, psychological health (stress, anxiety, and depression), and physical health.

**Results:**

This study has been reviewed and approved by the University of New South Wales Human Research Ethics Committee (HC230506). This project will be carried out according to the National Statement on Ethical Conduct in Human Research (2007). As of October 2025, we have enrolled 18 participants and completed their data collection. Results are expected to be published in peer-reviewed journals during the first quarter of 2026. Participant confidentiality will be maintained in line with ethical considerations.

**Conclusions:**

This protocol reports methods to determine feasibility, acceptability, and preliminary health outcome data of the Healthy Body and Mind Program informed by individuals with lived experience of dementia and osteoarthritis.

**Trial Registration:**

ClinicalTrials.gov NCT06070818; clinicaltrials.gov/study/NCT06070818

**International Registered Report Identifier (IRRID):**

DERR1-10.2196/75816

## Introduction

### Background

Globally, approximately 55 million people are living with dementia [1]. Osteoarthritis is experienced by approximately 528 million people worldwide [2] and is associated with a 20% to 25% increase in dementia risk [3,4]. The prevalence of osteoarthritis has been reported in almost half of people living with dementia [5,6]. Both dementia and osteoarthritis are associated with increased pain and depression [7-9] and reduced quality of life (QoL) [8,10]. Comorbid conditions, such as osteoarthritis, depression, and dementia, exacerbate symptoms of a disease; for example, a higher number of comorbidities (ie, multimorbidity) correlates with more severe pain and depression [1].

Depression, anxiety, and reduced QoL are common in dementia [9,11] and osteoarthritis [12], in addition to pain [7]. Furthermore, osteoarthritis has been associated with a 1.2- to 1.5-fold increase in dementia risk [13] and rises to 5.7-fold when experienced with an additional chronic condition [14]. However, most older adults, including people experiencing cognitive decline, are living with comorbid conditions such as osteoarthritis [15,16]. Depression and anxiety are also commonly found as comorbid conditions along with cognitive decline [17] and osteoarthritis, and depression has also been linked with increased dementia risk [18].

Nonpharmacological lifestyle approaches involving physical activity (PA) have been shown to improve health outcomes for 26 different chronic conditions [19], including dementia [20] and osteoarthritis [7]. Osteoarthritis and other chronic conditions are associated with increased dementia risk [20,21], and PA and other nonpharmacological approaches improve health outcomes in older adults while reducing dementia risk [20]. PA interventions improve cognition in people living with dementia [22] as well as reduce dementia risk in older adults [20]. Studies have shown that PA and lifestyle approaches reduce dementia risk [14,20,23] and improve cognition and QoL for people already experiencing cognitive decline [24-26]. Furthermore, nonpharmacological approaches have been shown to be cost-effective [27] and preferable to pharmacological approaches for people living with dementia and their families and care partners [28].

Systematic reviews have shown that PA has a positive effect on cognition [29], depression, and QoL [30]. However, findings are inconsistent regarding the most effective type of PA. Multimodal and mind- and body-focused approaches tend to outperform singular or unimodal approaches [30]. Interventions shown to be effective in reducing pain in osteoarthritis include aerobic and strengthening exercises and multimodal approaches that incorporate lifestyle education specific to osteoarthritis, such as pain coping skills training [31]. Multimodal approaches have been shown to be more effective than unimodal approaches in reducing pain [7] and improving depressive symptoms. PA has positive effects on mental health [32] and improves outcomes for older adults living with anxiety and depression. Meta-analyses have shown that nonpharmacological approaches reduce symptoms of depression in people with cognitive decline [9] and osteoarthritis [7]. Nonpharmacological approaches include care staff education and reminiscence therapy for people living with dementia and multimodal exercise approaches (eg, combined strength, balance, aerobic, and mind-focused approaches such as tai chi and yoga) for people with osteoarthritis.

Despite this evidence, there is a lack of accessible, implementable, and scalable lifestyle programs for people living with comorbid conditions, such as cognitive decline and osteoarthritis. This gap reflects a major opportunity for improving health outcomes and reducing dementia risk. Guidelines for multidisciplinary approaches that include PA have been developed for people living with cognitive decline [33,34], but effective interventions are specifically needed to target people living with comorbidity [15,35] and reduce dementia risk. It is vital to consider associations between conditions and how symptoms may be exacerbated (eg, symptoms of depression resulting from limitations in completing usual daily activities due to osteoarthritis-related pain and cognitive difficulties).

### Objectives

**Figure 1:**
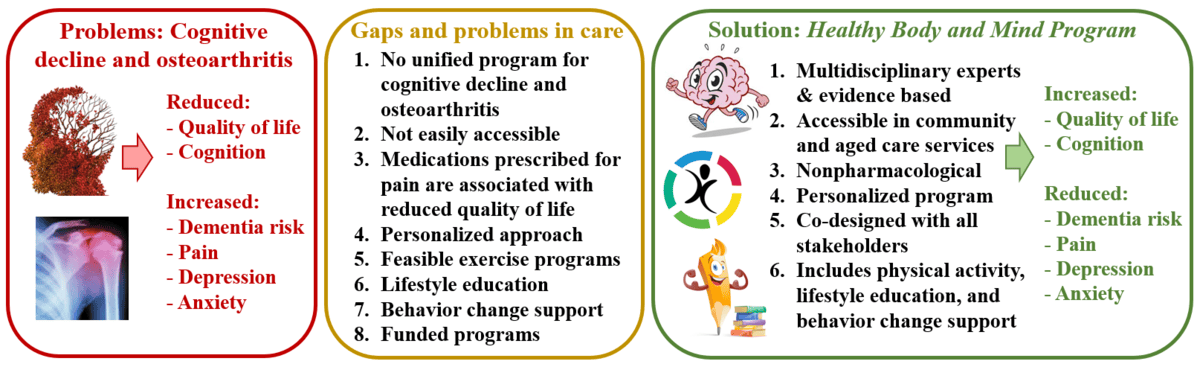
Evidence-based conceptualization illustrating how the Healthy Body and Mind Program will improve health outcomes for people with osteoarthritis and cognitive decline in community settings.

We will pilot the evidence-based Healthy Body and Mind Program (HBMP), which includes PA, nutrition, lifestyle, and behavior change education on health outcomes in people living with cognitive decline and osteoarthritis. The evidence base for development of the program is outlined in Figure 1. The primary aims of this pilot randomized controlled trial (RCT) are to determine (1) the feasibility (ie, recruitment, retention, and adherence) of the HBMP for people living with osteoarthritis and cognitive decline in a community clinic and the University of New South Wales (UNSW) Medicine and Health Lifestyle Clinic, Sydney, Australia, and (2) the acceptability of the program to people living with osteoarthritis and experiencing cognitive decline. The secondary aim is to obtain pilot data on health outcomes in people with osteoarthritis and cognitive decline, following the program when compared to usual care. Health outcomes are QoL, cognition, pain, psychological health (depression, anxiety, and stress), and physical health (weight, waist circumference, blood pressure, cardiorespiratory fitness, strength, mobility, and balance). We hypothesize that individuals who complete the program will have better health outcomes when compared to the baseline and the control groups.

## Methods

### Ethical Considerations

All data will be deidentified and participants will be allocated a unique identification number. Personal identifying information will be stored separately from research data in a password-protected file accessible only to the research team. Deidentified research data will be stored securely for a minimum of 7 years as per institutional requirements, after which it will be destroyed. Only deidentified data will be used in publications and presentations. All participants will provide written informed consent before participation, after reading the participant information sheet and having the opportunity to ask a researcher any questions. Participation is entirely voluntary and participants may withdraw at any time without penalty or impact on their access to services. Participants who withdraw can request that their data be destroyed, provided the data has not yet been deidentified and aggregated. All participants will receive a lifestyle program provided by accredited exercise professionals and psychologists free of charge (valued at approximately US $2000). Participants who additionally take part in the focus group will receive a voucher worth US $50 to compensate for the extra time commitment involved in this component of the study. All electronic data will be stored on password-protected, encrypted devices and secure university servers. Hard copy materials will be stored in locked cabinets in a secure research office. Only named members of the research team will have access to identifiable data. Ethics approval for this study has been obtained from the UNSW Human Research Ethics Committee (HC230506). This is the third version of the protocol and aligns with approved ethics amendments (most recently approved on June 14, 2024; first approval on September 18, 2023). Changes from the first version pertained to the inclusion criteria, where “Montreal Cognitive Assessment (MoCA) [36] score 18-25 indicating mild cognitive impairment or mild dementia” was removed, and the age requirement was reduced to people aged 45 years or older. These changes were made due to recruitment challenges, although they do not change the overall aims of the study. Other amendments included changes to the terminology used in study materials (“dementia,” “cognitive decline,” and “thinking difficulties” to improve recruitment) and updates to the list of staff members. All team members received email correspondence regarding submitted changes and were requested to review amended documentation where appropriate. The clinical trial has been prospectively registered on ClinicalTrials.gov (NCT06070818). This protocol has been prepared in accordance with the SPIRIT (Standard Protocol Items: Recommendations for Intervention Trials) guidelines (Multimedia Appendix 1) [37].

### Study Design

This 12-week pilot RCT of the HBMP with a waitlist control group will use quantitative and qualitative research methodologies. Data collection will comprise the following: (1) clinical observations routinely completed as part of standard care (eg, clinical interviewing, physical assessment, and exercise monitoring), (2) validated patient-reported outcome measures (including QoL, cognition, pain, and psychological health), and (3) focus group or interviews (Table 1). Clinical observations and questionnaires will be completed at baseline (0 wk) and after intervention (12 wk). Initial community and stakeholder engagement has assisted in developing the group program and ensuring it is feasible and acceptable to participants. The study team includes accredited exercise physiologists (AEPs), accredited practicing dietitians, a health psychologist, and research assistants (RAs). All study activities will take place within the UNSW Medicine and Health Lifestyle Community Clinic facilities.

**Table 1:** Data collection methods and outcome measures.

Measure or tool		Procedure (tools or guidelines)
**Clinical assessments (resting cardiovascular and anthropometry measures)**		
	Resting blood pressure and heart rate	Blood pressure will be measured using a validated automated sphygmomanometer. Participants will be seated and rested for at least 10 minutes before the blood pressure assessment [38]. Participants will place their forearm on a table with the elbow at an approximate 90° angle, and the cuff will be placed on the upper arm according to the manufacturer’s instructions. Three separate measurements will be taken on both right and left arms, with the average of the 3 measurements used in subsequent analyses.
	Waist circumference, height, weight, and BMI	Weight will be measured using a digital scale (SECA 813 model) with the participant unfasted, barefoot, and dressed in light clothing. Height will be measured barefoot using a wall-mounted stadiometer (SECA 216 stadiometer). Waist circumference will be measured according to the International Diabetes Federation guidelines [39].
	Handgrip strength	Handgrip strength will be measured using a manual dynamometer.
	Functional fitness	Functional fitness will be assessed using the submaximal 6-minute walk test [40]. The participant will be instructed to cover as much distance walking on flat ground in a laboratory setting until they have completed 6 minutes or they choose to stop walking. Risk of falls will be minimized by the AEP^a^ or RA^b^ following the participant closely to enable spotting and assistance should they lose balance. They will also monitor the participant throughout the test for safety (including heart rate and RPE^c^).
	Lower extremity function and mobility	Lower extremity function and mobility will be measured using the Short Physical Performance Battery [41], which involves timing the duration of 5 sit-to-stand repetitions from a chair, feet together, semitandem and full tandem stance, and a 2.44-m walk.
	Balance	Balance will be evaluated using the single-leg balance test. The participant will be instructed to stand on 1 foot for as long as possible without assistance up to a maximum of 60 seconds. To minimize the risk of falls, they will be supported by an AEP or RA, and there will be a wall or sturdy chair in front of them that they can hold onto if needed.
**Questionnaires**		
	APSS^d^	The APSS developed by Exercise and Sports Science Australia will be used to screen exercise readiness [42].
	Atherosclerotic Cardiovascular Disease Risk Estimator Plus	Cardiovascular disease risk will be estimated using the American College of Cardiology calculator [43].
	Active Australia Survey	Physical activity levels will be measured using the Active Australia Survey following the guide and manual for implementation, analysis, and reporting by the Australian Institute of Health and Welfare.
	Cognition (global, executive, learning, memory, and visuospatial ability)	Cognition will be assessed using the MoCA^e^ [36] and the Cambridge Neuropsychological Test Automated Battery (attention, memory, and learning tasks).
	Pain, physical health, and stiffness	Pain and stiffness will be evaluated using the WOMAC^f^ [44].
	Depression, anxiety, and stress	Psychological health will be assessed using the DASS-21^g^ [45].
	Quality of life	Quality of life will be measured using the SF-36^h^ (version 2) survey [46].
**Qualitative methods**		
	Participant feedback and recommendations	Participant feedback and recommendations will be obtained through focus groups or interviews using semistructured questions [47].
	Individual or stakeholder feedback and recommendations	Individual and stakeholder perspectives will be gathered through stakeholder meetings or workshops using a semistructured format.
Adverse events		Adverse events will be monitored and reported in accordance with CONSORT^i^ guidelines [48].

^a^AEP: accredited exercise physiologist.

^b^RA: research assistant.

^c^RPE: rating of perceived exertion.

^d^APSS: Adult Pre-Exercise Screening System.

^e^MoCA: Montreal Cognitive Assessment.

^f^WOMAC: Western Ontario and McMaster Universities Osteoarthritis Index.

^g^DASS-21: Depression, Anxiety, and Stress Scale-21.

^h^SF-36: Short Form Health Survey-36.

^i^CONSORT: Consolidated Standards of Reporting Trials.

### Sample Size Calculation

For pilot trials, using a 90% 1-sided CI, it is estimated that a sample size should be at least 9% of that calculated for a fully powered RCT [49]. Given a minimal clinically important difference of 0.5 SD (medium effect size), QoL measured using the Short Form Health Survey-36 [50], α of .05 (with Holm-Šídák correction), and power of 90% (β=.20) for a 2-group comparison, the required sample size would be approximately 85 participants per group (170 total). Accounting for potential dropout or attrition (15%-20%), 102 participants per group (N=204 total) would need to be recruited for a fully powered trial, and 16 participants would be required for a pilot trial. This study aims to recruit 20 participants.

### Eligibility Criteria

Eligibility criteria will be as follows: (1) aged 45 years or older, (2) living with osteoarthritis diagnosed by a health care professional, (3) living with cognitive concerns or dementia, (4) able to safely undertake study assessments and complete exercise without assistance, and (5) have appropriate medical clearance to take part. Exclusion criteria will be as follows: (1) MoCA score of less than 18; (2) unwilling or unable to provide written informed consent and comply with study requirements; (3) inability to speak and understand English unless a translator is available; and (4) contraindications to exercise in accordance with American College of Sports Medicine (ACSM) guidelines (Multimedia Appendix 2) [51], such as symptomatic hernias, proliferative retinopathy, uncontrolled arrhythmias, or rapidly progressing or terminal illness. Consent will be obtained by the lead researcher (CVB).

### Recruitment

A total of 20 participants with osteoarthritis and cognitive decline will be recruited through the UNSW Medicine and Health Lifestyle Clinic, StepUp for Dementia Research, and general practitioner and health district referrals. Study advertisements will also be displayed electronically via online social media platforms (eg, X, formerly known as Twitter [X Corp]; Facebook [Meta Platforms, Inc]; and LinkedIn [LinkedIn Corporation]). Initial contact will be made by potential participants emailing the study investigators through the study email or the StepUp team or by referral from a health professional. A member of the research team will then contact potential participants. Participants will be screened in person, online, or over the telephone to confirm their eligibility. The lead investigator will review participant screening data and confirm participant eligibility before providing the participant information sheet and consent form (Multimedia Appendix 3). If eligible and after written consent has been obtained, potential participants will be invited to an in-person screening visit. Demographic information, chronic disease history, lifestyle behaviors, and medication use will be documented. Potential participants will complete the Adult Pre-Exercise Screening System and Exercise and Sports Science Australia [42] screening questionnaires for exercise-induced symptoms (eg, chest pain and musculoskeletal pain; Multimedia Appendix 4). To ensure safety, only people cleared through the screening will be enrolled and randomized. Once deemed eligible, participants will complete written informed consent, and baseline testing will commence. Participants will be assigned a unique ID number to ensure personal details are not linked to collected data.

Due to ethical considerations where many potential participants may not be aware of cognitive decline, the term “thinking difficulties” will be used throughout study advertisements and participant information sheets. An ethically approved protocol is in place for advising people of their health assessment results (eg, cognition and depression) and referring them to their general practitioner where appropriate. Eligible participants will have mild cognitive impairment; therefore, they will have the capacity to provide informed consent.

Following successful screening, the participant will be allocated a unique ID number using the generated list. The RA who performs the randomization will advise the data collectors of the participants’ IDs by referring to the password-protected spreadsheet of participant names and allocated ID numbers. After completion of baseline testing, participants will be randomly assigned to the intervention (n=10) or waitlist control groups (n=10) using a computer-generated randomization list produced by a researcher external to the study group. In total, 20 participants will complete the program. After completing a 12-week period of usual care, those in the waitlist control group will then complete the 12-week HBMP. A 12-week follow-up will be performed to examine sustainability. Participants are allowed to withdraw from the trial at any time they wish and are assured that doing so would not alter their care or relationship with the university or the community clinic.

### Data Collection and Outcome Measures

#### Feasibility and Acceptability

The feasibility of the program will be determined by the number of eligible participants who provide consent and retention and adherence to the program (ie, number of exercise and education sessions attended, completed, and engaged with). Retention will be determined by the percentage of participants who provide consent and complete the program. Adherence will be determined by the percentage of program sessions attended by participants who enter the program. Adherence will be recorded using attendance records (recorded by the AEP and health psychologist facilitating the sessions). The acceptability of the program will be assessed by postprogram evaluation questionnaires after the program is completed.

#### Health Outcomes

Baseline and postintervention assessment visits will take between 90 and 120 minutes, including the health interview, physical assessment, and questionnaire completion. The RA completing assessments will be blinded to the condition (intervention or waitlist control groups). The RA will be trained and supervised by an AEP.

Secondary outcome measures will be completed before randomization at baseline and 12 weeks after intervention. The outcome measures (questionnaires outlined in the subsequent section) will be completed by an RA and a member of the research team who is blinded to the group allocation. Health outcome measures will include QoL (measured using the Short Form Health Survey-36) [46]; cognition (MoCA [36] and the Cambridge Neuropsychological Test Automated Battery attention, learning, and memory tasks); pain (Western Ontario and McMaster Universities Osteoarthritis Index) [44,52]; psychological health (Depression, Anxiety, and Stress Scale-21) [45]); and physical health (weight, waist circumference, blood pressure, functional fitness, strength, mobility, and balance). Balance exercises will be simple easy to perform movements that take place in a purposefully designed environment that minimizes the risk of falls (ie, they will be beside a wall, with an AEP closely monitoring them and simple exercises will be completed first, enabling the AEP to continually assess whether it is safe for the participant to continue to the slightly more challenging balance exercises).

The questionnaires (Table 1) will assess the participants’ health relevant to the outcome measures (eg, cognition, QoL, and psychological health). The data collection process will be administered using UNSW Qualtrics, except for the cognition measures, as these require the participant to draw on the printed tool (ie, pen and paper questionnaire). Participants will be asked questions by an experienced health psychologist and RA who has experience interviewing people with cognitive decline. They will enter the data directly onto a tablet or paper for the MoCA (with no identifying information, just a participant ID). The MoCA data will then be entered electronically. The health psychologist has received appropriate training to administer the MoCA [36] and a license (obtained in July 2022) and permission to train other staff and students to administer the tool. Participants will be invited to bring a support person with them if they wish, given that variability in level of independence is expected in this population (people with osteoarthritis and cognitive decline). All questionnaires have been validated and used extensively in the literature and with clinical populations, including people with osteoarthritis and cognitive decline.

### Data Management

The questionnaires will be completed within the UNSW Medicine and Health Lifestyle Clinic facilities. The data captured on Qualtrics and the Cambridge Neuropsychological Test Automated Battery software using a computer or tablet will be exported to Microsoft Excel and SPSS Statistics (IBM Corp) for data analysis and stored on a secure OneDrive (Microsoft Corporation) database hosted by UNSW, Sydney. The data will be entered, checked, and cleaned by 2 members of the research team. Only members of the research team will have access to the data. All recruited participants will be assigned a unique study ID number, ensuring no personal details, such as their names, are stored alongside data.

### Adverse Events

PA may cause concern in older adults and people living with chronic conditions. Adverse events may occur (eg, a fall), despite the safety protocols we have put in place (eg, AEP or RA closely monitoring participants during exercise in a purposefully designed clinic or laboratory setting with handrails). Throughout the trial and follow-up, adverse events will be documented, and risk will be continually assessed by appropriately trained professionals (ie, AEP). Reported adverse events will include details about the type of event, date, time, and duration; severity (mild to severe); whether it could have been avoided; outcome (solved to fatal); and whether it was related to the study (certainly, probably, possibly, unlikely, or cannot say). The relevant ethics committee and participant’s general practitioner will be informed about any serious event (eg, requiring hospitalization) that takes place during the study. Adverse events will be reported transparently alongside study outcomes. In accordance with the CONSORT (Consolidated Standards of Reporting Trials) statement [48] and similar to other studies [53], data from participants who are suspended from the trial due to an adverse event will be entered into the analysis by their allocated treatment group. The lead researcher will continue to communicate and support the participant and signpost them to relevant support services where appropriate. An independent safety monitoring process has been established (the data safety monitoring board) to oversee participant safety and review this trial. These arrangements have been documented in a safety monitoring plan, reviewed and approved by the human research ethics committee. All research staff involved in this trial have completed training and certification in Good Clinical Practice guidelines.

### Intervention

#### Overview

The HBMP has been designed in line with the strongest evidence for dementia risk reduction [20,21] and treatment for osteoarthritis [7,31,54]. Furthermore, the exercise component was designed according to international guidelines for prescribing exercise in older adults from the ACSM [51]. Health behavior theories that informed the intervention were the health belief model [55] and behavior change techniques [56]. The health belief model focuses on how individuals perceive health threats (eg, aging, pain, dementia, and decreased mobility) and the likelihood that actions taken toward their goals will lead to success. Behavior change techniques include shaping knowledge, providing information on the health consequences of behavior, action planning, self-monitoring of behavior, graded tasks, and social support. Participants will focus on goals, barriers, and enablers using the specific, measurable, achievable, relevant, and time-bound goals approach [57]. Taken together, the program aims to promote participants’ health behavior changes. Measures of behavior change, such as knowledge and self-efficacy, are not being included in this pilot feasibility trial, although following successful completion of the pilot, they will be incorporated into a fully powered RCT.

The program will be delivered within the UNSW Medicine and Health Lifestyle Clinic facilities by AEPs and health or clinical psychologists. The 12-week program sessions will take place twice per week (24 sessions in total) and will be 60 to 90 minutes long, including exercise, lifestyle, and behavior change education. Both weekly sessions will comprise 45 minutes of supervised exercise. The first weekly session will be followed by 45 minutes of education (including pain self-management, nutrition, sleep, and reducing smoking and alcohol consumption). Participants will be assigned to time slots suitable for them at the beginning of the program to help reduce the number of missed sessions. Catch-up sessions will be available for participants who are unable to attend their allocated slot. Four groups (2-8 participants per group) will complete the HBMP for 6 months (2 groups in a waitlist control group for the first 3 months). Participants can choose the days and times that suit them and will repeat those for the full 12-week program.

#### Exercise Component

The exercise component will be evidence based and delivered using a variety of exercise equipment available within the UNSW Medicine and Health Lifestyle Clinic facilities as well as through independent exercise sessions completed at home. Exercises will be modified for each individual based on ability and fitness. A combination of aerobic, resistance, balance, and flexibility exercises will be prescribed to suit the needs of the individual participants.

The ACSM guidelines state that older adults should aim to complete “resistance training twice a week and aerobic exercise of moderate intensity for 150-300 minutes a week, or vigorous intensity for 75-150 minutes per week” [51,58]. Therefore, the exercise prescription guidelines presented in Table 2 will be followed. Participants will complete resistance training twice weekly at the clinic in addition to 60 minutes of aerobic activity and 90 minutes or more of independent or home-based aerobic activity. Independent activity will be recorded each week on a printed record sheet and returned to the AEP upon completion. This will total the ACSM’s recommended 150 minutes per week of aerobic activity and 2 strength-based sessions that older adults should engage in for health benefits (Table 2) [51,58]. The prescriptive elements of all exercises performed will be recorded in program training sheets for each session to ensure all necessary details are captured. The education sessions will provide information on how participants can complete PA independently (Table 3).

**Table 2:** Healthy Body and Mind Program—clinic-based exercise component.

	Aerobic exercise	Resistance exercise
Frequency	Twice weekly (clinic based)	Twice weekly (clinic based)
Duration	30 minutes	30 minutes
Intensity	Light to vigorous (light: RPE^a^ 8-10; moderate: RPE 11-13; vigorous: RPE 14-16) [59]	Light to vigorous (light: 30%-49% 1 RM^b^; RPE 9-11; moderate: 50%-69% 1 RM; RPE 12-13; vigorous: 70%-84% 1 RM; RPE 14-17)
Type	Major muscle groups: cycling (upright or recumbent), walking (treadmill), rowing, and stepping	Pin-loaded weight plate machines (eg, leg press, leg extension, leg curl, seated row, chest press, and latissimus [or “lat”] pull down), body weight, free weights, and resistance bands
Volume	Frequency×intensity×time	Sets×repetitions×days; 1 to 3 sets and 8 to 12 repetitions
Pattern	One continuous bout or multiple bouts (eg, 30 min or 3×10 min)	Rest between sets and exercises
Progression	Commence first exercise session in week 1 at low to moderate intensity (50%-60% HRmax^c^; RPE 9-13) and progress gradually as tolerated by the individual	Increased weights to ensure participants’ RPE remains within the required range

^a^RPE: rating of perceived exertion [59].

^b^RM: repetition maximum.

^c^HRmax: maximum heart rate (beats per min).

**Table 3:** Healthy Body and Mind Program—lifestyle and behavior change education component in accordance with leading organizations.

Session topic^a^	Resource (fact sheets or guidelines) and organizations
Personalized goals and action plans	Behavior change techniques, action planning, and SMART^b^ goals
Nonpharmacological approaches	Osteoarthritis (Arthritis Australia); mild cognitive impairment (Dementia Australia)
Physical activity	Osteoarthritis fact sheet (Exercise is Medicine Australia); physical exercise and dementia (Dementia Australia)
Pain neuroscience education	UNSW^c^ Medicine and Health; Arthritis NSW^d^; Exercise is Medicine Australia; Musculoskeletal Australia
Nutrition	Healthy eating and arthritis (Arthritis Australia); food diary
Psychological health	Arthritis and emotional well-being (Arthritis Australia); depression and dementia fact sheet (Dementia Australia)
Social connections	Social connectedness in older adults (Centre for Healthy Brain Ageing, UNSW); American Association of Retired Persons
Sleep	Arthritis Foundation; Sleep Foundation; Musculoskeletal Australia
Alcohol and smoking	Arthritis Foundation; Australian Government Department of Health; American Heart Association
Participant’s choice	Participants allowed to choose areas they would like to focus on

^a^Each session was facilitated by educational slides developed by CVB.

^b^SMART: specific, measurable, achievable, relevant, and time bound.

^c^UNSW: University of New South Wales.

^d^NSW: New South Wales.

The AEP and health psychologist will receive training from 2 of the study investigators (CVB and BJP) to deliver the exercise and education components. Exercise monitoring observations will take place to record and assess the participants’ compliance with the prescribed exercise program relevant to the secondary outcome measures. This data collection will be undertaken face-to-face during group exercise sessions. The AEP will conduct the clinical assessment and group exercise sessions. Each session’s data will be collected using program sheets already used in the clinic to capture descriptive details of specific exercises. Measurements of heart rate, blood pressure, and rating of perceived exertion (Borg Rating of Perceived Exertion [59]) will be recorded during the group sessions. These data will be analyzed to provide information on the overall intensity of exercise achieved in each group session.

#### Lifestyle and Behavior Change Education Component

The education component will also take place at the UNSW Medicine and Health Lifestyle Clinic and involve group discussions developed by a health psychologist and delivered by a health and clinical psychologist. The education topics and resources are presented in Table 3. In summary, they include information about living with osteoarthritis and cognitive decline, PA, nutritional advice, psychological health, social connections, developing individual goals and action plans, and final sessions determined by individual or group preferences. Educational materials are presented in the resources column of Table 3 and have been developed by organizations, including Dementia Australia, Arthritis New South Wales, Alzheimer’s Association, and the Australian Government Department of Health. Printed and electronic copies will be provided to participants and used to guide discussions.

### Statistical Analysis and Clinical Interpretation

Feasibility, acceptability, and preliminary efficacy of the program will be analyzed. Feasibility will be examined by assessing group session attendance, quantified as total sessions attended out of a maximum possible and reported as a percentage. Feasibility will be quantified as session compliance with criteria for success, requiring performance of at least 10 minutes of aerobic training and 5 progressive resistance training exercises per session. Participant attendance, exercise programming, and adverse events will be recorded. Reasons for nonattendance will be collected and reported. Adverse events will be recorded, and ongoing monitoring and reporting will be completed in accordance with CONSORT guidelines [48]. Acceptability will be determined by participants’ perspectives and experiences of the program based on final participant feedback (focus groups or interviews). Acceptability will encompass what participants liked most and least about the program and their suggestions for the content and format of the sessions. Preliminary efficacy of the program will involve analysis of health outcomes (QoL, cognition, and psychological health). Quantitative data will be analyzed using SPSS Statistics (version 30.0.0.0, 172), with normality checks and descriptive statistics for frequencies and distributions. Baseline data will be compared between control and intervention groups to ensure there are no significant differences between groups at baseline. Health outcomes will be compared pre- and postprogram completion and between the intervention and control groups using 1-tailed *t* tests and repeated measures ANOVAs. Statistical significance will be set to *P*<.05. Effect sizes will be calculated using Cohen *d* for *t* tests and ηp^2^ for ANOVAs. Results will be reported comparing the intervention versus the waitlist control group and before versus after the 12-week program. Qualitative data will be analyzed using thematic content analysis [47].

### Dissemination

The project findings will be submitted to national and international conference presentations and scientific journal publications. Findings will also be prepared appropriately for other stakeholder groups (eg, lay summaries for people with lived experience of chronic conditions, families, and the public and reports for health care, government, and industry partners). When participants provide consent, they will be invited to indicate whether they would be interested in receiving study findings. Preliminary findings from this project have been presented at national and international conferences (eg, the Australian Dementia Research Forum and the Alzheimer’s Association International Conference). The outcomes will be published in high-quality peer-reviewed scientific journals.

### Patient and Public Involvement

The design of the program was informed by meetings with people living with osteoarthritis and dementia. WY is a study investigator and is a dedicated professional in the dementia and health care community. They currently serve as a living experience associate consultant at HammondCare, are a member of the Alzheimer’s Disease International (ADI) Global Review Panel for Accreditation, the Public Involvement Panel for StepUp for Dementia Research, and the Sleep, Lifestyle, Engagement, and Activity Program (SLEAP). They have been a member of the Dementia Australia Advocacy Program since 2020. In addition, they hold the role of vice chair at Dementia Alliance International and are part of the Forward with Dementia co-design group. Their professional engagement includes participation in numerous international and national conferences, such as the ADI Global Conferences in Krakow and London; the ADI Asia-Pacific Conference in Taiwan; and various Australian conferences, including the International Dementia Conference and Australian Dementia Research Forum. They have also established their own website, awakeningyourpositivity.com, to further share their insights and expertise.

WY is a lead investigator on this project and has been involved in all stages of developing this research. Prestudy consultation identified specific considerations for people living with osteoarthritis and experiencing cognitive decline and highlighted different experiences and expectations of a program such as the HBMP. In summary, feedback included practical considerations, such as transport and accessibility to take part in the program (eg, building and environmental considerations, ease of access, and lighting), the option of having a support person while participating in the program, ensuring the program included multiple components though considering that individuals may differ in how much support they would like to receive from each component (eg, nutritional advice sought by some and considered less important by others), including a social component to the program or considering social factors, and ensuring a tailored approach is adopted to suit participants’ unique needs. We have set up a community lived experience group of 30 people within our clinic who were invited to provide feedback on this study at the beginning of the project development. Participants and support persons will be invited to take part in a focus group or interview at the end of the pilot RCT to provide their feedback and share their experiences. These meetings and focus groups will inform the interpretation and dissemination of findings and guide future large-scale implementation.

## Results

As of October 2025, we have enrolled 18 participants and completed their data collection. Results are expected to be published during the first quarter of 2026.

## Discussion

### Anticipated Findings

This is a pilot RCT with complementary quantitative and qualitative components that aims to determine the feasibility and acceptability of a 12-week HBMP. Furthermore, we will examine whether the program improves QoL, cognition, and other health outcomes (pain, depression, and physical health) in people with osteoarthritis and cognitive decline. Currently, there is no program available to meet the specific needs of this population, despite the prevalence of dementia rapidly increasing and the fact that half of older adults globally are living with chronic pain, often attributable to osteoarthritis. The program has been developed in partnership with people with lived experience of dementia and osteoarthritis, AEPs, clinical researchers, chronic disease specialists, and accredited practicing dietitians. The program will continue to be codeveloped, ensuring it is feasible in a real-world setting and easily scalable.

The anticipated findings of this study are that the 12-week program will be feasible and acceptable for people living with osteoarthritis and thinking concerns. Participant feedback will be valuable in informing a larger clinical trial. Regarding secondary outcome measures, we hypothesize that improvements will be observed for pain, QoL, physical function, and cognition in those who complete the program, though these may not be significant given the small sample size.

### Implications and Comparisons to Prior Work

This is the first program that has been developed to target osteoarthritis and thinking concerns, both of which are specific risk factors associated with dementia. Currently, people are not aware that they can reduce their dementia risk [60], and societal awareness of dementia is lacking [61] even among health care professionals. The World Alzheimer Report [60] found that 62% of the health care professionals and 70% of the general population surveyed (across 155 countries) think dementia is a part of normal aging. Alarmingly, 25% believe that nothing can be done to prevent dementia [60]. This is particularly concerning given the overwhelming evidence demonstrating that approximately 45% of dementia cases could be prevented or delayed if modifiable risk factors, such as those that form the focus of this program (ie, physical inactivity and obesity), were eliminated [20], highlighting an opportunity for universal dementia prevention. With regard to interventions for osteoarthritis, medicines can have serious adverse side effects and have been associated with increases in addiction, morbidity, and mortality [62]. Many studies have demonstrated positive effects on pain, QoL, and depression following PA [7,63], dietary interventions [64], pain education [65], and other lifestyle behaviors. However, none have combined effective PA and lifestyle education into a single program as we have done here, nor have the effects on outcomes associated with dementia, such as cognition, been investigated in this higher-risk group.

### Strengths and Limitations of This Study

The first stage of the study involved conducting interviews with people with lived experience of osteoarthritis and dementia to inform the design of the HBMP. Their recommendations were incorporated into the final design of the program**.** In the second stage, participants have been recruited to complete a pilot RCT. Feasibility and acceptability will be examined. Health outcomes, including QoL, pain, cognition, psychological and physical health, will be compared before and after intervention and between program and control groups. Following the program, participants will take part in interviews or focus groups to determine the acceptability of the program and investigate the views of participants, including their recommendations for future programs. In the third stage, stakeholder workshops with individuals, health care providers, and policymakers will take place to discuss future implementation of similar programs at a larger scale with broader outreach. Recruitment and feasibility challenges may impact the success of the trial. Mitigation strategies include considering the language used in recruitment materials, advertising the trial widely using multiple recruitment platforms and health care contacts, and ensuring smooth communication processes between researchers and participants.

Strengths of this study are the range of health outcomes being investigated (eg, QoL, pain, cognition, and psychological measures), allowing for in-depth inquiry into multiple factors associated with comorbidity and dementia risk. The qualitative component will provide feedback from experts living with osteoarthritis and cognitive decline regarding whether the program was enjoyable and feasible and met their needs as well as providing an opportunity to gather recommendations to improve the program and deliver it on a larger scale to benefit more people.

A multicomponent, evidence-based program that has been informed by diverse experts, including those with lived experience, may improve health outcomes for older adults with osteoarthritis and cognitive decline and reduce dementia risk. This is timely, given the substantial increases in dementia cases predicted globally.

## Appendix

Multimedia Appendix 1 SPIRIT (Standard Protocol Items: Recommendations for Intervention Trials) checklist.

Multimedia Appendix 2 Contraindications to exercise.

Multimedia Appendix 3 Participant information sheet and consent form.

Multimedia Appendix 4 Pre-exercise screening questionnaires.

## Abbreviations

ACSM: American College of Sports Medicine
ADI: Alzheimer’s Disease International
AEP: accredited exercise physiologist
CONSORT: Consolidated Standards of Reporting Trials
HBMP: Healthy Body and Mind Program
MoCA: Montreal Cognitive Assessment
PA: physical activity
QoL: quality of life
RA: research assistant
RCT: randomized controlled trial
SPIRIT: Standard Protocol Items: Recommendations for Intervention Trials
UNSW: University of New South Wales

## Data Availability

The datasets generated or analyzed during this study are available from the corresponding author on reasonable request.
