# Supplementary material for: Healthy Body and Mind Program to Improve Health Outcomes and Reduce Dementia Risk in People With Osteoarthritis: Protocol for a Feasibility and Acceptability Pilot Randomized Controlled Trial
Source: JMIR Res Protoc. 2025 Nov 6;14:e75816. doi: 10.2196/75816 (PMC12635591; doi:10.2196/75816)
Supplement: Multimedia Appendix 2 [file resprot_v14i1e75816_app2.pdf]

Appendix 2. Contraindications to exercise adapted from ACSM guidelines.

American College of Sports Medicine (ACSM). ACSM's guidelines for exercise testing and prescription: Lippincott Williams & Wilkins; 2013, page 118.

Absolute Contraindications

- Acute myocardial infarction within 2 days.
- Ongoing unstable angina.
- Uncontrolled cardiac arrhythmia with hemodynamic compromise.
- Active endocarditis.
- Symptomatic severe aortic stenosis.
- Decompensated heart failure.
- Acute pulmonary embolism, pulmonary infarction, or deep venous thrombosis.
- Acute myocarditis or pericarditis.
- Acute aortic dissection.
- Physical disability that precludes safe and adequate testing.

Relative contraindications

- Known obstructive left main artery stenosis.
- Moderate to severe aortic stenosis with uncertain relationship to symptoms.
- Tachyarrhythmias with uncontrolled ventricular rates.
- Acquired advanced or complete heart block.
- Recent stroke or transient ischemia attack.
- Cognitive impairment with limited ability to follow instructions.
- Resting hypertension with systolic >200 mm Hg or diastolic >110 mm Hg.
- Uncorrected medical conditions, such as significant anemia, important electrolyte imbalance, and hyperthyroidism.
