# Supplementary material for: Healthy Body and Mind Program to Improve Health Outcomes and Reduce Dementia Risk in People With Osteoarthritis: Protocol for a Feasibility and Acceptability Pilot Randomized Controlled Trial
Source: JMIR Res Protoc. 2025 Nov 6;14:e75816. doi: 10.2196/75816 (PMC12635591; doi:10.2196/75816)
Supplement: Multimedia Appendix 3 [file resprot_v14i1e75816_app3.pdf]

PARTICIPANT INFORMATION STATEMENT AND CONSENT FORM

Healthy Body & Mind Program for older adults living with osteoarthritis and thinking difficulties.

Lead Investigator: Dr Claire Burley

1. **What is the research study about?**
Thank you for your interest in this research study. The research study aims to evaluate the effects of a Healthy Body & Mind Program on health outcomes including cognition, quality of life and pain, for older adults living with osteoarthritis and thinking difficulties in a real-world setting.

2. **Who is conducting this research?**
The study is being carried out by the following researchers:

Role	Name	Organisation
Chief Investigator	Dr Claire Burley	School of Health Sciences & School of Clinical Medicine, UNSW Sydney
Co-investigator/s	A/Prof Belinda Parmenter<br>Dr Matthew Jones<br>Prof Henry Brodaty<br>Dr Nattai Borges	School of Health Sciences, UNSW Sydney<br>School of Health Sciences, UNSW Sydney<br>School of Clinical Medicine, UNSW Sydney<br>School of Health Sciences, UNSW Sydney
Associate Investigator	Mr Bill Yeates	Community Member
Research and clinical staff	Ms Kimberley Au<br>Ms Sara Asadi<br>Ms Mia Ryan<br>Ms Stephanie Tsang	School of Health Sciences UNSW Sydney<br>School of Health Sciences UNSW Sydney<br>School of Health Sciences UNSW Sydney<br>School of Health Sciences UNSW Sydney

Research Funder: This research is being supported by philanthropic funding provided by Faye Williams and a research grant awarded from the UNSW Neuroscience, Mental Health & Addiction (NHMA) Theme, UNSW Sydney.

3. **Inclusion/Exclusion Criteria**
Before you decide to participate in this research study, we need to ensure that it is fine for you to take part. The research study is looking to recruit people who meet the following criteria:
    - Adults aged 45 or above.
    - Montreal Cognitive Assessment score of 18 or above.
    - Living with osteoarthritis.
    - Able to safely undertake study assessments and complete exercise without assistance.

    Participants who meet the following criteria will be excluded from the study:
    - Montreal Cognitive Assessment score below 18.
    - Display an abnormal cardiovascular response to exercise (as seen during the baseline exercise test) and your doctor does not provide medical clearance to exercise.
    - Are unable to speak English and do not have a translator.
    - Have orthopedic or neuromuscular limitations that prevent you from performing the exercise.
    - Are deemed unsuitable for group exercise sessions following initial assessment by an Accredited Exercise Physiologist within the UNSW Medicine & Health facilities.

Do I have to take part in this research study?
Participation in this research study is voluntary. If you do not want to take part, you do not have to. If you decide to take part and later change your mind, you are free to withdraw from the study at any stage.

If you decide you want to take part in the research study, you will be asked to:

- Read the information carefully (ask questions if necessary).
- Sign and return the consent form if you decide to participate in the study.
- Take a copy of this form with you to keep.

4. What does participation in this research require, and are there any risks involved?

If you agree to participate you will be asked to complete the research procedures described below. In summary they include two visits to the UNSW Medicine & Health facilities at the beginning and end of the program for Initial and Final Assessments, and two weekly sessions for 12 weeks (24 sessions in total) to the UNSW Medicine & Health facilities to complete the Healthy Body & Mind Program (see figure below). You will also be invited to take part in a focus group either at the clinic or online (via Teams/ zoom) at the end of the program.

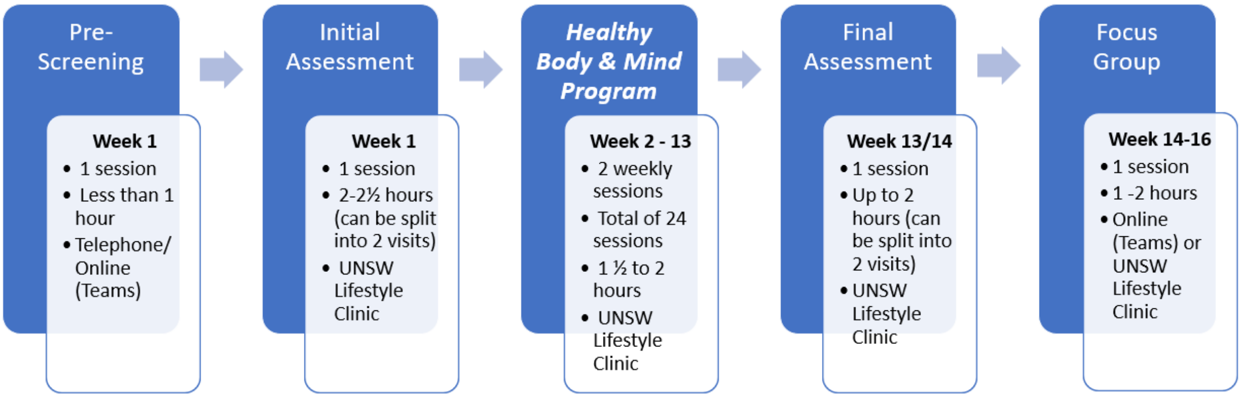

Initial and Final Assessments:

You will first undergo an initial assessment (2-2½ hours, this can be split into 2 visits) with a researcher including questionnaires, a pre-exercise screen, a clinical interview (including medical and lifestyle history) and clinical assessments. Each of these are part of standard clinical practice at the UNSW Medicine & Health facilities. You will then be randomly assigned to take part in either (1) the first 12-week group or (2) a 12-week wait-list control, where the program will start 12 weeks later. You can continue your usual care and activities during this period.

The clinical interview will include questions about your health and physical activity history. The final assessments are expected to be up to 2 hours (this can be split into 2 visits), as a brief medical update will replace initial clinical interview. The researcher will use some clinical screening questionnaires to assess your starting exercise levels and cardiovascular risk. The questionnaires you undertake will assess your physical activity levels, quality of life, cognition, pain levels, social networks, sleep, diet, self-efficacy and physical activity enjoyment and mood.

Diet: We will record your diet over 24 hours using a self-complete computerised dietary recall program called Intake24.

Resting Cardiovascular Measures: Resting blood pressure and heart rate will be measured using an automated cuff. You may feel brief pressure around your arm as the blood pressure cuff inflates, this may be a little uncomfortable, but will not cause any harm.

Body Composition: We will measure your height, weight, and waist circumference. We do not expect any of these tests to cause any harm or discomfort. These tests will take approximately 5 minutes to complete.

Strength Assessment: The strength tests will measure the maximal weight that you can push and pull for one repetition with your arms and legs (1RM). You will perform three tests for your upper and lower body. We will also measure your hand grip strength.

Functional fitness will be using a 6 Minute Walk Test. You will be instructed to cover as much distance walking until 6 minutes is up or your heart rate reaches 85% of your aged-predicted heart rate maximum. Your heart rate and rating of perceived exertion will be monitored throughout the test for safety.

Balance and functional mobility will be assessed with the Short Physical Performance Battery which involves timing your duration to perform 5x sit-to-stands from a chair, standing with feet together, semi-tandem and full tandem stance and an 8-foot walk. Balance will be assessed using a single leg balance test where you will be instructed to stand on one foot for as long as possible without assistance, up to a maximum of 60 seconds.

Cognition, quality of life, depression, anxiety and social networks: These are measured using validated questionnaires and computerised tasks that are widely used in clinical and research settings. These will be delivered by a psychologist or trained student/research assistant. They will take approximately 30 minutes to 1-hour complete. You will be informed of the results of these measures. With your permission, we may share the results with your General Practitioner if the results indicate further investigation is recommended.

Healthy Body & Mind Program:
Following initial assessment, you will receive 24 group sessions (3-8 people per group) at a frequency of twice per week. Each session will run between 1 and 2 hours and include either educational (approximately 30 minutes), exercise (approximately 60 minutes), social engagement/psychological health (approximately 30 minutes) components.

The Accredited Exercise Physiologist will provide an evidence-based exercise program delivered utilising the variety of exercise equipment available within the UNSW Medicine & Health facilities. A combination of aerobic, resistance, balance and flexibility exercises will be prescribed as is suitable and relevant to you. The exercise prescription will follow the internationally accepted American College of Sports Medicine guidelines and each session your exercise program and performance will be monitored and recorded (including your heart rate, blood pressure and subjective rating of perceived exertion). Education sessions will include a range of topics to help manage osteoarthritis and thinking difficulties with a healthy lifestyle. Psychological health sessions will include topics focused on managing anxiety, depression and stress. Social engagement sessions will be relaxed group sessions with healthy drinks and snacks with a different theme each week (e.g., favourite place visited).

Following your final assessment, you will be invited to attend a focus group within 4 weeks. The 45-60 minutes focus group will be conducted online via Microsoft Teams, at a place and time convenient for

you (e.g., your home or other location with internet connection). If you do not have online access, you may be invited to attend the focus group from an available space within the UNSW Medicine & Health facilities. If you do need to attend face-to-face, all necessary physical distancing requirements will be adhered to. The focus group will involve two researchers guiding a recorded group discussion around your feedback regarding the Healthy Body & Mind Program, including what you enjoyed, disliked and suggestions for improvements. The session will be both audio and video recorded and transcribed. You may participate in the focus group with audio alone by choosing to join with call without a camera/video function. During the transcription process your details (including name and any other identifying features) will not be transcribed from the recordings.

As UNSW Medicine & Health operate learning and teaching facilities, patients are advised that Exercise Physiology undergraduate students complete their clinical placement and work alongside the Accredited Exercise Physiologists to deliver clinical services. All students involved in the project will have undergone an induction to the UNSW Medicine & Health facilities, be trained by the research team and be added to the ethics application prior to being involved in the project.

There is a small risk of muscle soreness or injury during the exercise testing and training programs. There is also a possible risk of injury or a heart attack during exercise, but these are very small, and the initial assessment and exercise testing will have minimised this risk further by screening you for cardiovascular disease and identifying your suitable starting exercise intensity. To minimise these risks, we will carefully monitor you throughout your training, prescribe a training program in accordance with your physical capabilities and take care to set up the exercise equipment in a manner to maximise your safety.

During each testing procedure, and at regular intervals throughout the training program, we will ask you to inform us of any side effects that you may experience. An Accredited Exercise Physiologist will supervise the exercise session and you can address any adverse events or questions you may have in any session with them. However, it is important that you contact the researchers (Claire Burley – 9065 3510 or c.burley@unsw.edu.au) immediately if there are any unusual health experiences, injury or bad effects, whether or not you believe this problem is related to the exercise program.

If you experience discomfort or feelings of distress while participating in the research, you can stop participating at any time. You can also tell a member of the research team and they will provide you with assistance or alternatively a list of support services and their contact details are provided below.

Psychological Distress: You may feel that some of the questions we ask are stressful or upsetting. If you do not wish to answer a question, you may skip it and go to the next question, or you may stop immediately. If you become upset or distressed as a result of your participation in the research project, the research team will be able to arrange for counselling or other appropriate support. Alternatively, a number of free contactable support services are included in section 11. Any counselling or support will be provided by qualified staff who are not members of the research team. This counselling will be provided free of charge.

5. What are the possible benefits of taking part?

You will receive a multidisciplinary evidence-based program free of charge (usual cost ~$508). You will also be reimbursed a $50 gift voucher for your time and participation in the focus group.
We cannot guarantee or promise that you will receive any benefits from this research; however, the program has been co-designed with people living with chronic conditions and developed from scientific research showing the most effective approaches. Possible benefits may include improved health and wellbeing from participating in the program. We also hope that your participation will help us better guide other older adults living with osteoarthritis and thinking difficulties in the future. We will provide you with a thorough health report after initial testing, as well as a report once you have completed the 12-week program.

6. What are the alternatives to taking part in the research?

You do not have to take part in this research project to receive treatment at the UNSW Medicine & Health facilities. As this research project is investigating the feasibility and acceptability of a real-world group clinical service for older adults living with osteoarthritis and thinking difficulties, the group service is yet to be delivered as a standard part of the UNSW Medicine & Health services. If you chose to not participate in the project, other options are available; such as attending the UNSW Lifestyle Clinic for other individualised Exercise Physiology services. The research team will be able to direct your queries to the clinic to discuss these options with you before you decide whether to take part in this research project. You can also discuss the options with your local doctor.

7. What will happen to information about me?

By signing the consent form, you consent to the research team collecting and using information about you for the research study.

The research team will store the data collected from you for this research project for:

- A minimum of 15 years after the publication of research results;

The information about you will be stored in an/a:

- Re-identifiable format where any identifiers such as your name, address, date of birth will be replaced with a unique code.

You will be asked to provide your consent for the research team the share or use the information collected from you in future research that:

- Will be used in any future research.

Your information will only be shared in a format that will not identify you.

- Information collected from you in an electronic format stored on a UNSW password protected OneDrive only accessible to the approved research investigators.
- Information collected from you using paper-based measures will be stored in the following: UNSW Medicine & Health facilities, Randwick NSW, and only the approved research investigators will have access to this information.

The information you provide is personal information for the purposes of the Privacy and Personal Information Protection Act 1998 (NSW). You have the right of access to personal information held about you by the University, the right to request correction and amendment of it, and the right to make a complaint about a breach of the Information Protection Principles as contained in the PPIP Act.

Further information on how the University protects personal information is available in the UNSW Privacy Management Plan.

8. How and when will I find out what the results of the research study are?

The research team intend to publish and report the results of the research. All Information will be published in a way that will not identify you. Published results will also be made available on the UNSW Lifestyle Clinic website. If you would like to receive a copy of the results you can let the research team know by inserting your email or mailing address in the consent form. We will only use these details to send you the results of the research.

9. What if I want to withdraw from the research study?

If you do consent to participate, you may withdraw at any time. You can do so by completing the 'Withdrawal of Consent Form' which is provided at the end of this document or you can ring the research team and tell them you no longer want to participate. Your decision not to participate or to withdraw from the study will not affect your relationship with UNSW Sydney, the UNSW Medicine & Health facilities or any of the organisations involved in this research. If you decide to leave the research study, the researchers will not collect additional information from you. You can request that any identifiable information about you be withdrawn from the research project.

10. What if I have a complaint or any concerns about the research study and will I receive compensation if suffer any injuries or have complications?

If you suffer any injuries or complications as a result of this research project, you should contact the study team as soon as possible and you will be assisted with arranging appropriate medical treatment. If you are eligible for Medicare, you can receive any medical treatment required to treat the injury or complication, free of charge, as a public patient in any Australian public hospital.

Complaints Contact

If you have a complaint regarding any aspect of the study or the way it is being conducted, please contact the UNSW Human Ethics Coordinator:

**Position**	UNSW Human Research Ethics Coordinator
**Telephone**	+ 61 2 9385 6222
**Email**	humanethics@unsw.edu.au
**Ethics Number**	HC230506

11. What should I do if I have further questions about my involvement in the research study?
The person you may need to contact will depend on the nature of your query. If you require further information regarding this study or if you have any problems which may be related to your involvement in the study, you can contact the following member/s of the research team:

Senior Research Officer

**Name**	Kimberley Au
**Position**	Senior Research Officer
**Telephone**	[Removed from publication]
**Email**	k.au@unsw.edu.au

Chief Investigator Contact Details

**Name**	Dr Claire Burley
**Position**	Chief Investigator
**Telephone**	02 9065 3510
**Email**	c.burley@unsw.edu.au

Support Services Contact Details
If at any stage during the study, you become distressed or require additional support from someone not involved in the research please call:

**Name/Organisation**	National Arthritis and Back Pain+ Helpline, Musculoskeletal Australia
**Position**	Free telephone supporter
**Telephone**	1800 263 265<br>If you require an interpreter, please call the Translating and Interpreting Service on 131 450<br>If you are deaf or have a hearing or speech impairment, please call the National Relay Service on 1800 263 265
**Website**	https://msk.org.au/get-supported/

**Name/Organisation**	Lifeline
**Position**	Crisis supporter
**Telephone**	13 11 14
**Website**	https://www.lifeline.org.au/

**Name/Organisation**	Beyond Blue
**Position**	Crisis supporter
**Telephone**	1300 22 4636
**Website**	https://www.beyondblue.org.au/

BLANK PAGE

	UNSW SYDNEY

PARTICIPANT INFORMATION STATEMENT AND CONSENT FORM

Healthy Body & Mind Program for older adults living with osteoarthritis and thinking difficulties.

Lead Investigator: Dr Claire Burley

Consent Form – Participant providing own consent

Declaration by the participant

- ☐ I understand I am being asked to provide consent to participate in this research study;
- ☐ I have read the Participant Information Sheet, or someone has read it to me in a language that I understand;
- ☐ I understand the purposes, study tasks and risks of the research described in the study;
- ☐ I have had an opportunity to ask questions and I am satisfied with the answers I have received;
- ☐ I freely agree to participate in this research study as described and understand that I am free to withdraw at any time during the study and withdrawal will not affect my relationship with any of the named organisations and/or research team members;
- ☐ I provide my consent for the information collected about me to be used for the purpose of future research;
- ☐ I would like to receive a copy of the study results via email or post, I have provided my details below and ask that they be used for this purpose only;
- ☐ I understand that I will be given a signed copy of this document to keep.
- ☐ I understand that the results of the research will be made available on the UNSW Lifestyle Clinic website.
- ☐ I would like to receive a copy of the study results via email or post, I have provided my details below and ask that they be used for this purpose only.

Name: _______________________________________

Address: _____________________________________

Email Address: ________________________________

Optional Consent for reuse of data and future research:

- ☐ I provide my consent for the information collected about me to made available to other researchers as described at section 7 of this document.
- ☐ I provide my consent for my name and contact details to be retained in a register so I can be contacted about other research projects in the future.

Participant Signature

Name of Participant (please print)	
Signature of Research Participant	
Date	

Declaration by Researcher*

☐ I have given a verbal explanation of the research study, its study activities and risks and I believe that the participant has understood that explanation.

Researcher Signature*

Name of Researcher (please print)	
Signature of Researcher	
Date	

+An appropriately qualified member of the research team must provide the explanation of, and information concerning the research study. All parties signing the consent section must date their own signature.

	UNSW SYDNEY
**PARTICIPANT INFORMATION STATEMENT AND CONSENT FORM**<br>*Healthy Body & Mind Program* for older adults living with osteoarthritis and thinking difficulties.<br><br>Lead Investigator: Dr Claire Burley	

Form for Withdrawal of Participation

I wish to **WITHDRAW** my consent to participate in this research study described above and understand that such withdrawal **WILL NOT** affect my relationship with The University of New South Wales, and the UNSW Medicine & Health facilities.

- ☐ I am withdrawing my consent to participate in further components of this research and provide my permission for the research team to retain and/or use information collected about me which I have provided for the purpose of this research.

Participant Signature

Name of Participant (please print)	
Signature of Research Participant	
Date	

The section for Withdrawal of Participation should be forwarded to:

CI Name:	Dr Claire Burley
Email:	c.burley@unsw.edu.au
Phone:	+61 2 9065 3510
Postal Address:	School of Helath Sciences, Level 2 Wallace Wurth Buidling, UNSW Sydney, NSW 2052
