# Supplementary material for: Healthy Body and Mind Program to Improve Health Outcomes and Reduce Dementia Risk in People With Osteoarthritis: Protocol for a Feasibility and Acceptability Pilot Randomized Controlled Trial
Source: JMIR Res Protoc. 2025 Nov 6;14:e75816. doi: 10.2196/75816 (PMC12635591; doi:10.2196/75816)
Supplement: Multimedia Appendix 4 [file resprot_v14i1e75816_app4.pdf]

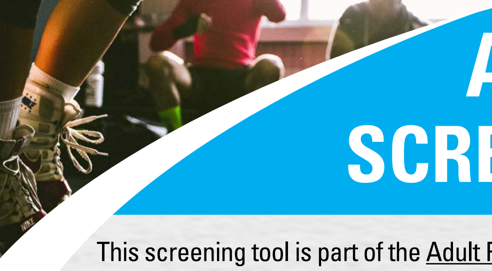

ADULT PRE-EXERCISE SCREENING SYSTEM (APSS)

This screening tool is part of the Adult Pre-Exercise Screening System (APSS) that also includes guidelines (see User Guide) on how to use the information collected and to address the aims of each stage. No warranty of safety should result from its use. The screening system in no way guarantees against injury or death. No responsibility or liability whatsoever can be accepted by Exercise & Sport Science Australia, Fitness Australia, Sports Medicine Australia or Exercise is Medicine for any loss, damage, or injury that may arise from any person acting on any statement or information contained in this system.

Full Name: ______________________________

Date of Birth: ____________________ Male: ☐ Female: ☐ Other: ☐

STAGE 1 (COMPULSORY)

AIM: To identify individuals with known disease, and/or signs or symptoms of disease, who may be at a higher risk of an adverse event due to exercise. An adverse event refers to an unexpected event that occurs as a consequence of an exercise session, resulting in ill health, physical harm or death to an individual.

This stage may be self-administered and self-evaluated by the client. Please complete the questions below and refer to the figures on page 2. Should you have any questions about the screening form please contact your exercise professional for clarification.

Please tick your response	YES	NO
1. Has your medical practitioner ever told you that you have a heart condition or have you ever suffered a stroke?		
2. Do you ever experience unexplained pains or discomfort in your chest at rest or during physical activity/exercise?		
3. Do you ever feel faint, dizzy or lose balance during physical activity/exercise?		
4. Have you had an asthma attack requiring immediate medical attention at any time over the last 12 months?		
5. If you have diabetes (type 1 or 2) have you had trouble controlling your blood sugar (glucose) in the last 3 months?		
6. Do you have any other conditions that may require special consideration for you to exercise?		

IF YOU ANSWERED 'YES' to any of the 6 questions, please seek guidance from an appropriate allied health professional or medical practitioner prior to undertaking exercise.

IF YOU ANSWERED 'NO' to all of the 6 questions, please proceed to question 7 and calculate your typical weighted physical activity/exercise per week.

7. Describe your current physical activity/exercise levels in a typical week by stating the frequency and duration at the different intensities. For intensity guidelines consult figure 2.

**Intensity**	**Light**	**Moderate**	**Vigorous/High**
**Frequency** (number of sessions per week)	______	______	______
**Duration** (total minutes per week)	______	______	______

Weighted physical activity/exercise per week

Total minutes = (minutes of light + moderate) + (2 x minutes of vigorous/high)

TOTAL = ________ **minutes per week**

- If your total is less than 150 minutes per week then light to moderate intensity exercise is recommended. Increase your volume and intensity slowly.
- If your total is more than or equal to 150 minutes per week then continue with your current physical activity/exercise intensity levels.

- It is advised that you discuss any progression (volume, intensity, duration, modality) with an exercise professional to optimise your results.

I believe that to the best of my knowledge, all of the information I have supplied within this screening tool is correct.

Client signature: ______________________ Date: ______________

FIGURE 1: Stage 1 Screening Steps

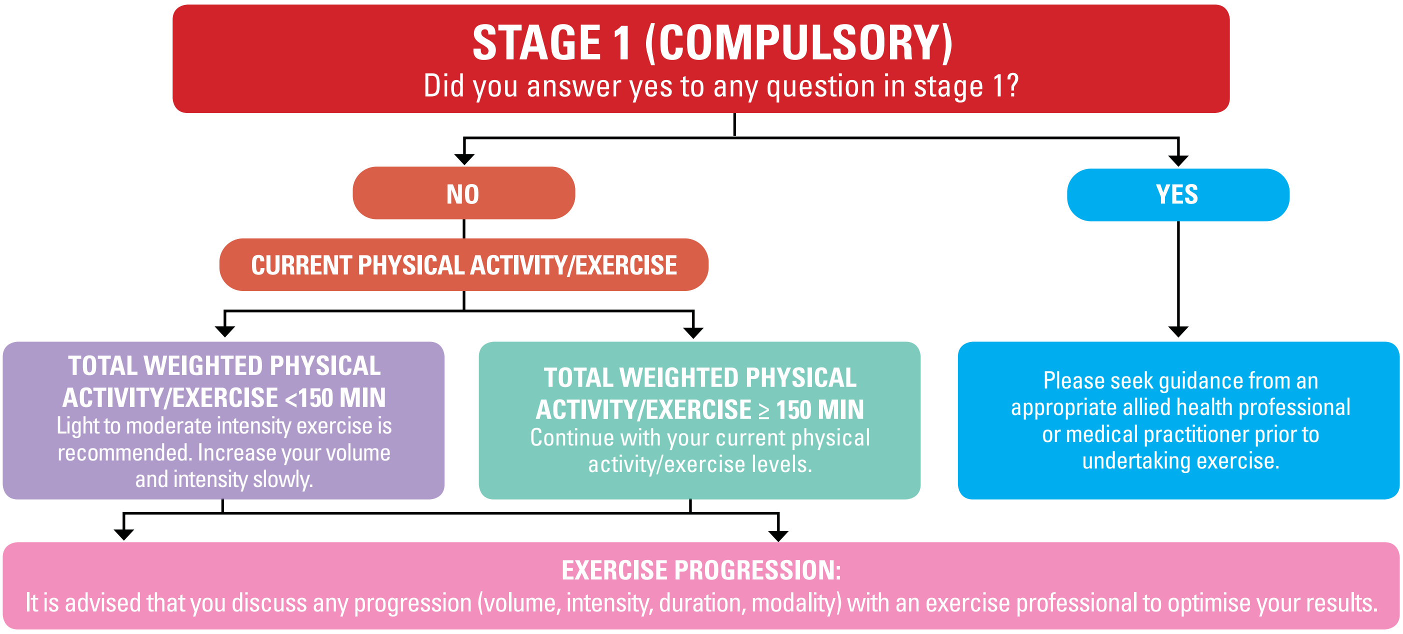

FIGURE 2: Exercise Intensity Guidelines

INTENSITY CATEGORY	HEART RATE MEASURES	PERCEIVED EXERTION MEASURES	DESCRIPTIVE MEASURES
LIGHT	40 to <55% HRmax*	VERY LIGHT TO LIGHT RPE# 1-2	• An aerobic activity that does not cause a noticeable change in breathing rate<br>• An intensity that can be sustained for at least 60 minutes
MODERATE	55 to <70% HRmax*	MODERATE TO SOMEWHAT HARD RPE# 3-4	• An aerobic activity that is able to be conducted whilst maintaining a conversation uninterrupted<br>• An intensity that may last between 30 and 60 minutes
VIGOROUS	70 to <90% HRmax*	HARD RPE# 5-6	• An aerobic activity in which a conversation generally cannot be maintained uninterrupted<br>• An intensity that may last up to 30 minutes
HIGH	≥ 90% HRmax*	VERY HARD RPE# 7	• An aerobic activity in which it is difficult to talk at all<br>• An intensity that generally cannot be sustained for longer than about 10 minutes

* HRmax = estimated heart rate maximum. Calculated by subtracting age in years from 220 (e.g. for a 50 year old person = 220 - 50 = 170 beats per minute).

# = Borg's Rating of Perceived Exertion (RPE) scale, category scale 0-10.

Modified from Norton K, L. Norton & D. Sadgrove. (2010). Position statement on physical activity and exercise intensity terminology. J Sci Med Sport 13, 496-502.

STAGE 2 (RECOMMENDED)

AIM: This stage is to be completed with an exercise professional to determine appropriate exercise prescription based on established risk factors.

CLIENT DETAILS	GUIDELINES FOR ASSESSING RISK
8. Demographics<br>Age: ____________<br>Male ☐ Female ☐ Other ☐	Risk of an adverse event increases with age, particularly males ≥ 45 yr and females ≥ 55 yr.
9. Family history of heart disease (e.g. stroke, heart attack)?<br>Relationship (e.g. father) Age at heart disease event<br>__________________ __________<br>__________________ __________<br>__________________ __________	A family history of heart disease refers to an event that occurs in relatives including parents, grandparents, uncles and/or aunts before the age of 55 years.
10. Do you smoke cigarettes on a daily or weekly basis or have you quit smoking in the last 6 months?<br>Yes ☐ No ☐<br>If currently smoking, how many per day or week?<br>________________________________	Smoking, even on a weekly basis, substantially increases risk for premature death and disability. The negative effects are still present up to at least 6 months post quitting.
11. Body composition<br>Weight (kg) ________ Height (cm) ________<br>Body Mass Index (kg/m^2) ________<br>Waist circumference (cm) ________	Any of the below increases the risk of chronic diseases:<br>BMI ≥ 30 kg/m^2<br>Waist > 94 cm male or > 80 cm female
12. Have you been told that you have high blood pressure?<br>Yes ☐ No ☐<br>If known, systolic/diastolic (mmHg)<br>______________________________<br>Are you taking any medication for this condition?<br>Yes ☐ No ☐<br>If yes, provide details<br>__________________________________	Either of the below increases the risk of heart disease:<br>Systolic blood pressure ≥ 140 mmHg<br>Diastolic blood pressure ≥ 90 mmHg
13. Have you been told that you have high cholesterol/ blood lipids?<br>Yes ☐ No ☐<br>If known:<br>Total cholesterol (mmol/L) ___________<br>HDL (mmol/L) ___________<br>LDL (mmol/L) ___________<br>Triglycerides (mmol/L) ___________<br>Are you taking any medication for this condition?<br>Yes ☐ No ☐<br>If yes, provide details _________________________	Any of the below increases the risk of heart disease:<br>Total cholesterol ≥ 5.2 mmol/L<br>HDL < 1.0 mmol/L<br>LDL ≥ 3.4 mmol/L<br>Triglycerides ≥ 1.7 mmol/L

CLIENT DETAILS	GUIDELINES FOR ASSESSING RISK
14. Have you been told that you have high blood sugar (glucose)?<br><br>Yes ☐ No ☐<br>If known:<br>Fasting blood glucose (mmol/L) ____________<br>Are you taking any medication for this condition?<br><br>Yes ☐ No ☐<br>If yes, provide details<br><br>____	Fasting blood sugar (glucose) ≥ 5.5 mmol/L increases the risk of diabetes.
15. Are you currently taking prescribed medication(s) for any condition(s)? These are additional to those already provided.<br><br>Yes ☐ No ☐<br><br>If yes, what are the medical conditions?<br><br>____	Taking medication indicates a medically diagnosed problem. Judgment is required when taking medication information into account for determining appropriate exercise prescription because it is common for clients to list 'medications' that include contraceptive pills, vitamin supplements and other non-pharmaceutical tablets. Exercise professionals are not expected to have an exhaustive understanding of medications. Therefore, it may be important to use common language to describe what medical conditions the drugs are prescribed for.
16. Have you spent time in hospital (including day admission) for any condition/illness/injury during the last 12 months?<br><br>Yes ☐ No ☐<br><br>If yes, provide details<br><br>____	There are positive relationships between illness rates and death versus the number and length of hospital admissions in the previous 12 months. This includes admissions for heart disease, lung disease (e.g., Chronic Obstructive Pulmonary Disease (COPD) and asthma), dementia, hip fractures, infectious episodes and inflammatory bowel disease. Admissions are also correlated to 'poor health' status and negative health behaviours such as smoking, alcohol consumption and poor diet patterns.
17. Are you pregnant or have you given birth within the last 12 months?<br><br>Yes ☐ No ☐<br><br>If yes, provide details<br><br>____<br>____<br>____	During pregnancy and after recent childbirth are times to be more cautious with exercise. Appropriate exercise prescription results in improved health to mother and baby. However, joints gradually loosen to prepare for birth and may lead to an increased risk of injury especially in the pelvic joints. Activities involving jumping, frequent changes of direction and excessive stretching should be avoided, as should jerky ballistic movements. Guidelines/fact sheets can be found here: 1) www.exerciseismedicine.com.au 2) www.fitness.org.au/Pre-and-Post-Natal-Exercise-Guidelines
18. Do you have any diagnosed muscle, bone, tendon, ligament or joint problems that you have been told could be made worse by participating in exercise?<br><br>Yes ☐ No ☐<br><br>If yes, provide details<br><br>____<br>____	Almost everyone has experienced some level of soreness following unaccustomed exercise or activity but this is not really what this question is designed to identify. Soreness due to unaccustomed activity is not the same as pain in the joint, muscle or bone. Pain is more extreme and may represent an injury, serious inflammatory episode or infection. If it is an acute injury then it is possible that further medical guidance may be required.

Important Information: This screening tool is part of the Adult Pre-Exercise Screening System ('APSS') and should be read with the APSS guidelines (see User Guide) on how to use the information collected and to address the aims of each stage. This does not constitute medical advice.This form, the guidelines and the APSS (together 'the material') is not intended for use to diagnose, treat, cure or prevent any medical conditions, is not intended to be professional advice and is not a substitute for independent health professional advice. Exercise & Sports Science Australia, Fitness Australia, Sports Medicine Australia and Exercise is Medicine (together 'the organisations') do not accept liability for any claims, howsoever described, for loss, damage and/or injury in connection with the use of any of the material, or any reliance on the information therein. While care has been taken to ensure the information contained in the material is accurate at the date of publication, the organisations do not warrant its accuracy. No warranties (including but not limited to warranties as to safety) and no guarantees against injury or death are given by the organisations in connection with the use or reliance on the material. If you intend to take any action or inaction based on this form, the guidelines and/or the APSS, it is recommended that you obtain your own professional advice based on your specific circumstances.
